# Targeting Mononuclear Phagocyte Receptors in Cancer Immunotherapy: New Perspectives of the Triggering Receptor Expressed on Myeloid Cells (TREM-1)

**DOI:** 10.3390/cancers12051337

**Published:** 2020-05-23

**Authors:** Federica Raggi, Maria Carla Bosco

**Affiliations:** Laboratory of Molecular Biology, IRCCS Istituto Giannina Gaslini, 16147 Genova, Italy; federicaraggi@gaslini.org

**Keywords:** mononuclear phagocytes, tumor-associated macrophages and dendritic cells, tumor microenvironment, cancer immunotherapy, pattern recognition and immunoregulatory receptors, triggering receptor expressed on myeloid cells

## Abstract

Inflammatory cells are major players in the onset of cancer. The degree of inflammation and type of inflammatory cells in the tumor microenvironment (TME) are responsible for tilting the balance between tumor progression and regression. Cancer-related inflammation has also been shown to influence the efficacy of conventional therapy. Mononuclear phagocytes (MPs) represent a major component of the inflammatory circuit that promotes tumor progression. Despite their potential to activate immunosurveillance and exert anti-tumor responses, MPs are subverted by the tumor to support its growth, immune evasion, and spread. MP responses in the TME are dictated by a network of stimuli integrated through the cross-talk between activatory and inhibitory receptors. Alterations in receptor expression/signaling can create excessive inflammation and, when chronic, promote tumorigenesis. Research advances have led to the development of new therapeutic strategies aimed at receptor targeting to induce a tumor-infiltrating MP switch from a cancer-supportive toward an anti-tumor phenotype, demonstrating efficacy in different human cancers. This review provides an overview of the role of MP receptors in inflammation-mediated carcinogenesis and discusses the most recent updates regarding their targeting for immunotherapeutic purposes. We focus in particular on the TREM-1 receptor, a major amplifier of MP inflammatory responses, highlighting its relevance in the development and progression of several types of inflammation-associated malignancies and the promises of its inhibition for cancer immunotherapy.

## 1. Introduction 

The onset of cancer involves a complex interplay among neoplastic, stromal, endothelial, and infiltrating inflammatory cells, which results in the establishment of a highly specialized tumor microenvironment (TME) [1]. Clinical and experimental evidence indicate that chronic inflammation is an indispensable participant in the neoplastic process, fostering genomic instability, epigenetic modifications, angiogenesis, cancer cell proliferation, survival, and dissemination [2,3,4,5,6,7]. Indeed, many cancers arise at sites of infection and chronic inflammation, and different inflammatory conditions, e.g., inflammatory bowel diseases (IBD), are highly correlated with the increased risk of neoplastic transformation [1,8,9,10]. Furthermore, cancer-related inflammation negatively affects the clinical efficacy of conventional therapies (chemotherapy and radiotherapy) and immunotherapy, antagonizing or hindering therapeutic responses [5,11]. 

The type of inflammatory cells present at tumor sites is responsible for tilting the balance between tumor progression and regression [1,3,5,12,13,14,15,16,17]. In particular, mononuclear phagocytes (MPs) have been recognized as major components of the inflammatory infiltrate in most solid human malignancies and crucial drivers of cancer-associated inflammation, being involved in every step of tumorigenesis from early transformation through to metastatic progression [3,4,5,18,19,20,21,22,23]. They are highly versatile immune cells able to adapt to different environmental conditions and display distinct phenotypes and functional programs dictated by a network of signals, including cytokines, microbial pathogens (pathogen-associated molecular patterns, PAMPs), molecules released by damaged/stressed cells (damage-associated molecular patterns, DAMPs), and metabolites [24,25,26,27,28,29,30,31,32,33]. Environmental stimuli are integrated through the cross-talk between multiple activatory/inhibitory receptor families, whose dynamic equilibria finely tune MP responses in diseased tissues, regulating their inflammatory and effector functions [34]. Alterations in receptor expression/activation can create excessive inflammation and, when chronic, promote tumorigenesis [33,35,36,37,38,39]. Given their role in carcinogenesis and influence on the effectiveness of anti-tumor therapies, MPs have attracted a lot of interest as potential targets of immunotherapeutic strategies, a concept that has already been investigated in several tumors [4,11,40,41,42,43].

In this review, we provide a comprehensive overview of published studies on MP physiopathology in the TME and an update of the state of the art of MP-targeted immunotherapeutic approaches. We summarize the current knowledge on the role of MP receptors in inflammation-mediated carcinogenesis and discuss the most recent advances regarding the attempts to their therapeutic targeting. We focus in particular on the triggering receptor expressed on myeloid cells (TREM1)-1, a major player in the amplification of MP inflammatory responses [44,45], highlighting its relevance in the development of several inflammation-associated malignancies and the promises of its inhibition as a novel therapeutic strategy in cancer. 

## 2. MPs in Tumors

### 2.1. MP Pro- and Anti-Cancer Activities 

MPs are recruited from the circulation to tumor sites by tumor-derived factors as primary monocytes (Mn), differentiating into tumor-associated macrophages (TAMs) or dendritic cells (TADCs) [4,18,19,20,46,47,48,49,50,51,52]. 

Macrophages are a heterogeneous cell population and a key component of innate defense mechanisms, exerting microbicidal and immunostimulatory activities. In the TME, TAMs display a dual influence on tumor progression [23,40,53,54]. They have the potential to activate immunosurveillance and exert anti-tumor responses by destroying cancer cells or inhibiting their proliferation through the release of cytokines, reactive oxygen species (ROS), and nitric oxide (NO), complement components, and prostaglandins. However, they can be subverted by the tumor to support its progression, spread, and immune evasion through the production of pro-angiogenic, mitogenic, metastatic factors, and immunosuppressive cytokines and the upregulation of inhibitory receptors [11,21,22,40,50,53,55,56]. Preclinical and clinical studies demonstrated that the nature of the activating stimulus and the combination of different stimuli in the TME can profoundly impact upon the type of response that occurs, polarizing TAMs into specialized functional subsets [24,26,30]. In addition, TAMs can undergo a rapid and reversible shift among functional programs in response to changes in the activating stimulus, often exhibiting mixed phenotypes [21,24,57,58,59,60]. It is currently accepted that TAMs involved in the early tumor initiation process display a “M1-like” pro-inflammatory and tumoricidal phenotype, activating Th1-type immune responses and eliminating transformed cells, but, as the tumor grows, they are educated by the TME to switch to an “M2-like” immunosuppressive and tumor-promoting phenotype, fostering tumor growth/metastatization and immune evasion [3,4,11,20,24,30,40,47,57,58,59,61,62]. High TAM infiltration in solid tumors is generally associated with poor prognosis and reduced overall survival in both experimental models and neoplastic patients [3,10,47,50,56,63,64,65,66,67], although a correlation with better prognosis has been suggested for some tumors [68]. 

DCs are professional antigen-presenting cells central to the orchestration of innate and acquired immunity and the maintenance of self-tolerance [51]. Deregulated DC responses may result in the amplification of inflammation, loss of tolerance, or establishment of immune escape mechanisms [25,33,69,70,71]. TADCs were described in the TME of many cancer types, and their inactivation was reported as one of the main mechanisms of tumor escape [72]. Several evidence suggest that TADCs can exist in a multitude of functional states during the course of the disease [46,71,73], and that their immunogenic capacity may be strongly conditioned by the TME, ranging from immunostimulatory to immunosuppressive [74,75]. In established tumors, TADCs display mostly an immature phenotype, characterized by a low expression of T-cell costimulatory and high levels of inhibitory molecules, defective migration to lymph nodes, and tolerance to tumor antigens, promoting tumor progression, dissemination, and immune evasion [46,48,73,74,76]. However, TADCs can generate tumor-specific adaptive immune responses, a capacity that is enhanced via DC-targeted vaccines [70,71,77]. 

### 2.2. Tumor Hypoxia Contributes to MP Pro-Tumoral Phenotype 

A critical hallmark of the TME, especially in advanced-stage tumors, is represented by low partial oxygen tension (pO_2_, 0–20 mm·Hg), referred to as hypoxia, which arises as a result of a disorganized or dysfunctional vascular network and poor O_2_ supply [78,79,80]. Hypoxia is an important driver of malignant progression, metastatic spread, and resistance to therapies and an indicator of poor prognosis in almost all solid tumors [13,78,81,82,83]. As documented by an extensive literature, hypoxia in the TME exerts multifaceted effects on every tumor component, influencing the nature and function of the inflammatory cell infiltrate and contributing to the establishment of immune resistance and tumor escape mechanisms [13,19,78,79,80,84,85,86,87,88,89,90,91]. 

Hypoxia is one of the critical signals regulating MP migration into tumors and conditioning the balance between their anti-/pro-tumoral functions [18,19,25,87,92,93,94]. Under hypoxic conditions, MPs are functionally reprogrammed through the differential expression of genes implicated in inflammation, angiogenesis, tissue disruption, mitogenesis, and immunoregulation [19,25,79,85,92,93,95]. Recent results point to the hypoxic environment as a direct trigger of human macrophage polarization towards a pro-tumoral “M2-like” state [31], confirming and extending studies in rodent tumor models showing that the intra-tumor O_2_ gradient is a critical regulator of the M1- to M2-skewed transition [61,93,96,97,98]. The correlation among the extent of M2-polarized TAM infiltration in hypoxic areas, tumor progression, and poor patient prognosis supports the hypothesis that reduced oxygenation contributes to MP acquisition of a pro-tumoral state [19,97]. Elucidation of the mechanisms underlying TAM/TADC dysregulated functions within the hypoxic TME may have important implications for their therapeutic reprogramming in tumors (see Section 2.3 for details).

### 2.3. Targeting MPs in Cancers 

Considerable efforts from several research groups have been dedicated to the development of anti-tumor immunotherapeutic strategies targeting MP recruitment to, and/or survival and functional polarization in, tumors [4,22,40,41,43,50,62,99]. Many studies have been carried out in experimental animal models, and a few drugs are currently under clinical trial investigation both as monotherapies or in combination with standard therapies [4,40]. 

The use of bisphosphonates encapsulated in liposomes or PEGylated nanoparticles to selectively deplete TAMs, owing to their phagocytic activities, showed promising anti-tumor effects in preclinical studies, reducing tumor burden, angiogenesis, and metastases. These agents are currently undergoing clinical trials as neoadjuvants in combination with chemotherapy and hormonal therapy [4,10,22,40,50]. Targeting the CSF1/CSF1R pathway, which is critical for Mn/macrophage survival and differentiation toward a M2 phenotype, with mAbs and small molecule inhibitors was used as an approach to neutralize immunosuppressive M2-like TAMs in tumors or induce their reprogramming toward a M1 phenotype and is being studied in phase I/II clinical trials. Several CSFR1 inhibitors demonstrated some anti-tumor response and reduction in tumor cell invasion, in particular, in combination regimens with conventional therapy or T cell-directed immunotherapy [4,10,11,22,40,43]. TAM accumulation in the tumor can be mediated by Mn recruitment through the CCL2–CCR2 axis, and CCL2 inhibition by specific Abs correlated with reduced TAM infiltration, tumor growth, and metastasis in various experimental models, alone or in association with chemotherapies, suggesting the efficacy of this approach [40,100,101]. Various CCL2-neutralizing Abs and a CCR2 inhibitor are now being tested in clinical trials, showing promises results [11,40,43,102,103]. TAM re-education from a pro-tumoral toward a pro- inflammatory/tumoricidal state was also proposed as a therapeutic strategy, eliminating the drawbacks and long-term toxicity of macrophage ablation. Immune checkpoint and/or anti-immunosuppressive cytokine inhibitors are currently being tested at both preclinical and clinical levels to boost TAM phagocytosis and effector functions or inhibit their immunosuppressive activity. Clinical trials combining anti-TAMs agents (such anti-CSF1R Abs) and immune checkpoint inhibitors are ongoing in different solid tumor contexts [4,43,104,105] (see Section 3.3 for details).

Promising developments in cancer-therapeutic strategies have also been made by targeting TADCs [72,106]. DCs have been used in vaccine preclinical models, and several phase I, II, and III clinical trials have tested the use of autologous Mn-derived DCs pulsed with tumor antigens to trigger anti-tumor T cell responses, with some results obtained in melanoma and prostate cancer patients [70,71,72]. Furthermore, TADC depletion in mice bearing ovarian cancer by targeting specific markers was also shown to significantly delay tumor growth and enhance the effect of standard chemotherapies [75]. More recently, the manipulation of TADCs to subdue their immunosuppressive functions and enhance their immune-stimulatory capacity has been carried out in preclinical studies, showing great promise [3,71,72,106,107] (see Section 3.3 for details).

Encouraging results obtained in preclinical studies and early clinical trials across various therapeutic modalities and tumor types highlight the possibility of translating MP-targeted immunotherapeutic strategies to the clinical practice to complement and improve the efficacy of current anti-cancer therapies [10,11,40,43].

## 3. MP Activatory/Inhibitory Surface Receptors 

### 3.1. Pattern Recognition Receptor (PRR) and Immunoregulatory Signaling (IRS) Receptors Expressed on MPs

Knowledge of the receptors regulating MP responses has largely increased in the past two decades [33,34,35,36,38,71,72,73,92,108,109,110,111,112,113,114,115]. The deregulated expression of various members of the scavenger/pattern recognition receptor (PRRs) and the inhibitory/activatory immunoregulatory signaling (IRS) receptor families in MPs has been reported to lead to aberrant inflammatory responses and trigger inflammatory diseases and inflammation-associated cancer development [71,72,73]. PRRs play a central role in the detection of and responses to PAMPs/DAMPs, triggering MP activation, immunogenicity, and pro-/anti-inflammatory and effector functions, which eventually result in pathogen clearance and tissue repair [33,38,39,71,114,116,117,118,119,120]. IRS receptors are involved in the pathogenesis of chronic inflammatory, allergic, and autoimmune diseases [34,35,92,108,112,120,121]. Their ligation can have both pro-inflammatory and immunoregulatory consequences by modulating differentiation/maturation, pro-/anti-inflammatory mediator secretion, phagocytosis, immune complex clearance, Ab-dependent cytotoxicity (ADCC), respiratory burst, and T cell priming [35,36,71,108,112,113].

### 3.2. Role of PRR and IRS Receptors in Tumors

**Figure 1:**
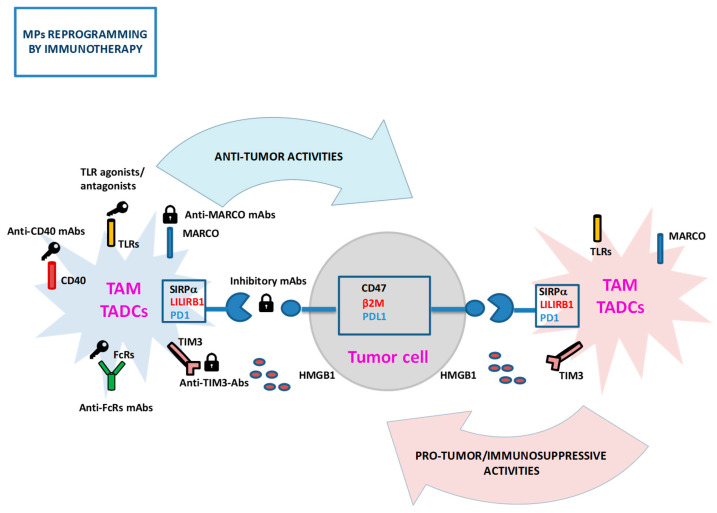
Schematic representation of pattern recognition receptor (PRR) and immunoregulatory signaling (IRS) receptors mediating mononuclear phagocytes (MP) pro-/anti-tumor activities and representing potential targets of cancer immunotherapy. The figure depicts a selection of MP receptors tested as potential immunotherapeutic targets in cancer. Targeting toll-like receptors (TLRs), CD40, Fc region of Igs (FcRs) activating receptors with agonist, or antagonist mAbs/ligands boosts tumor-associated macrophages (TAM)/tumor-associated dendritic cells (TADC) tumoricidal activities and T cell activation, enhancing their cytotoxicity, cancer cell phagocytosis, and/or antigen-presenting ability. Blockade of SIRPα LILRB1, PD-1 immunocheckpoint molecules, or macrophage receptor with collagenous structure (MARCO) and T cell Ig and mucin domain 3 (TIM-3) immunosuppressive receptors with inhibitory mAbs induces MP reprogramming toward an anti-tumor/immune-promoting state, eliciting TAM phagocytic activity and tumor cell killing and improving TADC ability to stimulate T cells. Activating and inhibitory approaches are indicated in the figure by keys and padlocks, respectively. TLRs, toll-like receptors; CD40, cluster of differentiation 40; FcRs, receptors for the Fc region of immunoglobulins; SIRPα, signal regulatory protein-alpha, CD47, cluster of differentiation 47; LILRB1, leukocyte immunoglobulin-like receptor B1; β2M, β2-microglobulin; PD-1, programmed cell death-1; PD-L1, PD-1 ligand; MARCO, macrophage receptor with collagenous structure; TIM-3, T cell Ig and mucin domain 3; HMGB1, high mobility group box 1.

Recent studies have highlighted the role of PRRs in mediating MP pro-/anti-tumor activities (Figure 1). Among them, toll-like receptors (TLRs) have been implicated in the regulation of macrophage polarization towards either an inflammatory/anti-tumor or a pro-tumorigenic phenotype in different cancers [4,99,122]. The expression of the macrophage scavenger receptor 1 (MSR1) was suggested to label a subset of anti-tumor TAMs, being significantly correlated with the inhibition of tumor progression, lower clinical stage, recurrence-free survival, and good prognosis in prostate cancer patients [64]. On the contrary, the macrophage receptor with collagenous structure (MARCO) was found expressed on a subtype of TAMs with an M2-like immunosuppressive phenotype in patients with mammary carcinoma, metastatic melanoma, and non-small cell lung cancer (NSCLC), and linked to poor prognosis [123,124]. 

Some IRS receptors have been shown to serve as immune checkpoints by inhibiting MP anti-tumor activation and favoring tumor immune escape [4,125,126] (Figure 1). The inhibitory receptor, signal regulatory protein-alpha (SIRPα), negatively regulates macrophage and DC phagocytic activity by interacting with its cognate ligand cluster of differentiation 47 (CD47) overexpressed on many types of cancer cells, increasing tumor invasion and metastasis [4,43,127]. Programmed cell death-1 (PD-1), as well as its ligand, PD-L1, are expressed on M2-like TAMs and TADCs (in addition to T lymphocytes) and upregulated during disease progression in both mouse cancer models and primary human tumors, inhibiting TAM phagocytic activity against tumor cells and the TADC adaptive immune-activating potential [72,74,104,106,128]. Another important immune checkpoint is represented by leukocyte immunoglobulin-like receptor B1 (LILRB1), which suppresses TAM phagocytic functions through the engagement of the MHC class I component β_2_-microglobulin (β_2_M) on cancer cells. The T cell Ig and mucin domain 3 (TIM-3) was also identified as an immune checkpoint expressed on a subset of macrophages, Mn, and DCs [129]. Its upregulation on DCs by factors present in the TME seems to be an important mechanism by which TADCs are locked into an immune-suppressive phenotype, preventing the detection of tumor-derived danger signals through an interaction with the DAMP molecule, high mobility group box 1 (HMGB1), and leading to the release of immune-suppressive factors, with consequent attenuation of the therapeutic efficacy of DNA vaccination and chemotherapy in experimental tumor models [130].

Elucidation of the stimuli in the TME involved in receptor deregulation has been the focus of intense research with the purpose of identifying new potential therapeutic targets. Hypoxic conditions similar to those present at tumor sites were reported to finely tune the PRR/IRS receptor repertoire in human Mn, Mn-derived macrophages, and DCs, by exerting a specific regulatory control on the expression profile of genes coding for various members of both receptor families, thus affecting cell responses toward an anti-tumor or a tumor-promoting direction [92,109,110,111,131,132,133,134].

### 3.3. PRR and IRS Receptors as Therapeutic Targets in Cancer 

Advances in understanding PRR/IRS receptor expression changes have led to promising developments in cancer-therapeutic strategies targeting TAM/TADCs to induce their switch from a tumor-supportive toward an anti-tumor phenotype (Figure 1). 

New immunotherapeutic approaches aimed at boosting TAM phagocytosis and effector functions using mAbs to specific receptors are currently under investigation [35,43,105]. Several therapeutic mAbs used in the clinic involve effector macrophages expressing the receptors for the Fc region of Igs (FcRs) to induce Ab-dependent phagocytosis (ADP) or ADCC of cancer cells (for a review see Reference [43]). Macrophage repolarization towards a tumoricidal phenotype was also attempted by targeting the TNF receptor superfamily member, CD40, which is expressed on MPs and binds CD40L on T cells [135]. Treatment with agonist anti-CD40 mAbs induced TAM immunostimulatory and tumor inhibitory effects in mouse tumor models both alone and in combination with anti-CSF1R mAb, by enhancing antigen-presentation and pro-inflammatory cytokine production and priming naive T lymphocytes [136,137,138,139]. This observation opened the way for the development of clinically relevant anti-CD40 mAbs, which have been tested in clinical trials for advanced-stage pancreatic tumors alone or in combination with chemotherapy, leading to partial response [4,43,140].

Another important example of Ab-based targeted therapy comes from studies of the CD47/SIRPα axis. Macrophages can be induced to phagocytize tumor cells by CD47/SIRPα-blocking agents, resulting in antigen presentation and promotion of adaptive immune responses against tumors [127]. Blocking SIRPα also polarizes TAMs to an “M1-like” anti-tumor phenotype [99]. Therapeutics targeting the CD47/SIRPα axis demonstrated anti-tumor efficacy both in vitro and in vivo in preclinical models and are currently being evaluated in clinical trials for both solid and haematologic malignancies as single agents and as combination therapies with tumor-opsonizing mAbs [4,43,126,127,141,142,143]. Blockade of the β2M/LILRB1 interaction was found to stimulate tumor cell phagocytosis by TAMs and significantly slow tumor growth in mice, representing an interesting anti-cancer immunotherapeutic approach [144]. Among the strategies aimed at TAM reprogramming, PD-1/PD-L1 targeting with specific mAbs was also shown to increase TAM phagocytosis, reduce tumor growth, and lengthen survival of tumor-bearing mice in a macrophage-dependent fashion [22,104,105]. Interestingly, high-dimensional profiling studies of immune cell populations infiltrating sarcomas of mice treated with anti-PD-1 mAbs, alone or in combination with anti-CTLA-4 mAbs, revealed Mn/macrophage remodeling in the tumor microenvironment, providing compelling support to the hypothesis that successful immune checkpoint therapy favors the generation of TAMs with an M1-like pro-inflammatory/anti-tumor phenotype while decreasing the induction of TAMs with an M2-like immunosuppressive phenotype [145]. Anti-MARCO mAbs similarly induced TAM re-education towards a pro-inflammatory phenotype in preclinical mouse models of breast and colon carcinoma and melanoma, resulting in increased tumor immunogenicity and tumor growth/metastasis inhibition, and improving the efficacy of checkpoint immunotherapy [123]. 

Another emerging approach to direct macrophage functions for cancer immunotherapy is to manipulate TLR signaling using synthetic ligands [99]. TLR3, TLR4, TLR7/8, and TLR9 agonists have been explored as vaccine adjuvants in different mouse cancer models, showing TAM repolarization toward an “M1 like” phenotype and improved tumoricidal activity both alone and in combination with immune-checkpoint inhibitors [146,147,148,149]. TLR7 and TLR9 agonists are currently being tested in clinical trials, resulting in tumor inhibition and increased lymphoid immune cell infiltration [4,99,122]. Anti-tumor properties of TLR2 and TLR4 antagonists or cognate ligands have also been observed [122]. 

TADC manipulation to rescue their immune stimulatory potential also represents an intriguing area of therapeutic impact [71,72,106]. Similarly to TAMs, TADCs are an important target of checkpoint inhibitor-based approaches, with promising results in preclinical studies. Blockade of the PD1/PD-L1 axis on murine TADCs improved their ability to stimulate T cell activation [72,74,128]. A recent study by Mayoux et al. [106] highlighted the strong correlation between a high DC gene signature and clinical response to treatment with an anti-PD-L1 mAb in patients with renal cell carcinoma and NSCLC. These investigators established the biological basis of PD-L1 blockade on TADCs, showing a disruption of the PD-L1/B7-1 interaction and CD28 costimulation by B7-1 on T cells, suggesting that anti-PD-L1 immunotherapy reinvigorates the TADC costimulatory function, enhancing T cell priming. TIM-3 targeting on TADCs by specific mAbs was also shown to delay tumor progression in a mouse model of lymphoma, although its efficacy is not completely clear and in part controversial due to the TIM-3 expression by multiple cell types with potentially different roles [129]. A number of anti-TIM3 mAbs are now being tested in early phase clinical trials as monotherapy or in combination with anti-PD-1/PD-L1 mAbs [150]. CD40 and TLR3 costimulation on TADCs was shown to increase their T cell immunostimulatory activity, inducing the rejection of ovarian carcinoma in a mouse model [151]. Recently, the combination of DC vaccines and checkpoint molecule inhibitors led to therapeutic responses in a phase II study in melanoma patients [3,72,107]. Furthermore, clinical use of TLR ligands as adjuvants for DCs vaccines against cancer has yielded promising results [72]. 

Taken together, these findings demonstrate that the use of Abs/ligands directed to specific MP receptors to shift their balance from a pro-tumorigenic/immunosuppressive toward an anti-tumoral phenotype represents an attractive alternative to classic tumor treatments, improving the efficacy of current immunotherapy in combinatorial strategies [22,105,122]. 

## 4. TREM 

Novel IRS receptors have been recently described in MPs. Among them, the TREM receptor family belonging to the Ig-like superfamily has been reported to participate in innate immune responses and be implicated in several infectious and non-infectious inflammatory diseases, autoimmune disorders, and cancers [44,152,153,154,155,156,157]. Six *trem* genes (*Trem1, Trem2, Treml1-4*) have been identified clustered on human chromosome 6p21 [153,158] and mouse chromosome 17C3, with four of them encoding structurally related type I transmembrane glycoproteins bearing a single extracellular Ig-like ectodomain (TREM-1, TREM-2, TREML-3, and TREML-4) [157,159]. The TREM isoforms have a short cytoplasmic tail (CYT) without a signaling motif, pairing for signaling with the transmembrane adapter, DNA-activating protein 12 (DAP12), which carries an immune receptor tyrosine-based activatory signal-transducing motif (ITAM) [152]. Despite similar structure and sequence homology, TREM isoforms show different cell-type expression patterns and functional activities [44,154,160]. TREM-1 (CD354) is the first identified and best-characterized family member and an important regulator of myeloid cell immune responses [37,45,153,154,161,162].

### 4.1. TREM-1 Structure and Expression Regulation in MPs

Two forms of TREM-1 were identified: 1) a transmembrane glycoprotein (≈30-kDa), composed of a signal peptide, the extracellular Ig-like domain, a membrane spanning region, and a short CYT [45,154,156,158,163,164,165]; 2) a soluble form (sTREM-1, ≈27 kDa), devoid of both transmembrane and cytoplasmic domains, either derived from alternative splicing of TREM-1 mRNA [153,155,166] or from shedding of the extracellular domain of the membrane-bound form by matrix metalloproteinase (MMP)-mediated proteolytic cleavage [167,168]. sTREM-1 acts as a decoy receptor, sequestering the TREM-1 ligand and preventing its binding to membrane-bound TREM-1 and receptor activation [162,169]. 

**Figure 2:**
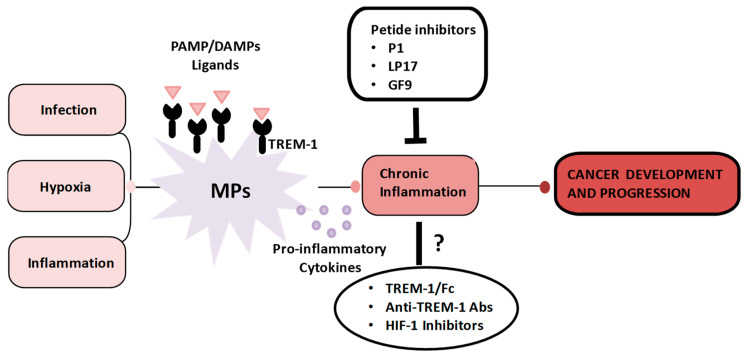
Role of TREM-1 expressed on MPs in chronic inflammation-associated carcinogenesis and potential as a new immunotherapeutic target in cancer. MPs are recruited to sites of inflammation, infection, and tumor growth, where they respond to microenvironmental stimuli, such as infectious/inflammatory agents and hypoxia, by upregulating TREM-1. TREM-1 engagement by specific pathogen-associated molecular pattern (PAMP)/damage-associated molecular pattern (DAMP) ligands present in the microenvironment promotes MP secretion of pro-inflammatory, chemotactic, angiogenic, and matrix-remodeling cytokines, resulting in the amplification of the ongoing inflammatory process and contributing to the development and progression of inflammation-associated malignancies. TREM-1 blockade by specific synthetic peptide inhibitors attenuates MP-mediated chronic inflammation and tumor progression in various preclinical mouse models, pointing to TREM-1 as a novel attractive target for cancer immunotherapy. Alternative TREM-1 inhibitors, such as the TREM-1/Fc fusion protein, antagonist anti-TREM-1 mAbs, and anti-HIF inhibition are currently being tested.

TREM-1 is developmentally regulated in MPs, being constitutively expressed in blood Mn and a subset of tissue macrophages [45,170] and downregulated upon Mn differentiation into DCs and Langerhans cells (LCs) [110,111,161,162,171,172,173]. Several stimuli can regulate TREM-1 expression (Figure 2). Increased TREM-1 surface levels and/or sTREM-1 release were demonstrated in vitro in both mouse and human Mn/macrophages in response to PRR activation by bacterial and viral PAMPs [37,45,168,174,175,176,177,178,179,180,181,182] and bacteria challenge [45,162,183], and in vivo in animals [162,168,182,184,185,186,187,188,189] and patients [162,181,185,190,191,192] suffering from bacterial, fungal, and viral infections. In addition, pro (TNFα)- and anti- (TGF-β, IL-10) inflammatory cytokines can increase and abrogate, respectively, TREM-1 expression [171,193]. Lipid mediators, such as prostaglandins, also modulate TREM-1 surface levels in both murine and human macrophages [194,195]. Expression regulation occurs mainly at the transcriptional level through the interaction of various transcription factors with specific sites in the TREM-1 promoter [37,183,196,197,198,199,200]. 

Interestingly, a major role for hypoxia as a TREM-1 trigger was demonstrated by Bosco and coworkers in distinct human MPs populations [31,109,110,111,201] (Figure 2). The TREM-1 transcript, membrane-bound protein, and sTREM-1 levels were significantly increased by hypoxia in primary Mn [109,202], Mn-derived macrophages [131], and upon M1/M2 polarization [31]. Furthermore, TREM-1 was induced ex novo on Mn-derived DCs [92,110,111] and LCs [201] generated under hypoxia, indicating that hypoxic stimulation can overcome TREM-1 developmental downregulation. A hypoxia response element (HRE) [25,78,203] was identified in the human TREM-1 gene promoter [110,111], and the hypoxia-inducible transcription factor (HIF)-1, the master regulator of cell response to hypoxia and an important activator of innate immune cells [204], was shown to be involved in TREM-1 inducibility by hypoxia [110,111]. TREM-1 expression was confirmed in vivo in macrophages, DCs, and LCs infiltrating hypoxic inflammatory tissues [31,110,201] and in TAMs infiltrating HIF-1+ glioblastoma and hepatocellular carcinoma (HCC) [202,205] (see Section 6 for details), suggesting the potential relevance of this molecule as a regulator of MP functions under hypoxic conditions.

### 4.2. TREM-1 Putative Ligands and Signaling 

The identification of the TREM-1 ligand remains controversial. A broad range of molecules have been proposed as putative TREM-1 ligands [37]. Studies with a recombinant TREM-1/Fc fusion protein, consisting of the human TREM-1 extracellular domain fused to the Fc portion of Igs, and anti-TREM-1 mAbs suggested the presence of a soluble ligand in the serum of septic patients or a membrane-bound ligand on the surface of human platelets, respectively [169,206]. HMGB1 and heat shock protein-70 have been proposed to act as TREM-1 ligands [207], and the interaction between TREM-1 and HMGB1 was demonstrated in a murine model [208]. Both the neutrophil-specific antigen, CD177, and the peptidoglycan recognition protein 1, a neutrophil granule protein with antibacterial properties, were also identified as potent TREM-1 ligands [157,209]. Finally, other PAMPs, such as the surface glycoprotein of Marburg and Ebola filoviruses [210], were recently included in the list of putative TREM-1 ligands.

TREM-1 engagement on the cell membrane triggers DAP12 association and Src family kinase-mediated ITAM tyrosine phosphorylation [45,154,156,161], followed by GRBP-2, Syk, and ZAP70 recruitment and phosphorylation, which initiate downstream signal transduction events. The signaling pathway mainly involves PI3K, PLC-γ, ERK1/2, p38 MAP, and Akt serine/threonine kinase activation [45,154,156,172], ultimately triggering intracellular Ca2+ mobilization, actin cytoskeleton rearrangement, and the activation of several transcription complexes [152,154,160], such as STAT5 and NF-κB [179]. The TREM-1 signaling pathway has been recently reviewed [37,179]. 

## 5. TREM-1 Role in Inflammatory Responses and Infectious/Non-Infectious Diseases

Since the TREM-1 discovery in 2000, extensive progress has been made regarding the elucidation of its biological effects and interaction with other receptor pathways. Due to the absence of a well-characterized ligand, TREM-1 functions have been studied using agonist mAbs, which induce receptor cross-linking. These studies demonstrated that TREM-1 is a key modulator of innate immunity to PAMPs/DAMPs and a major amplifier of MP inflammatory responses induced by PRRs [37,45,153,154,157,161,162,180] (Figure 2). TREM-1 triggering in human Mn was shown to drive the robust production of pro-inflammatory cytokines/chemokines and MMPs [45,171,211]. Microarray analysis showed the TREM-1 contribution to DC and LC pro-inflammatory reprogramming under hypoxic conditions, through the upregulation of genes coding for inflammatory cytokines/chemokines, angiogenic/growth factors, and MMPs [92,110,111,201]. These findings were extended by recent evidence that TREM-1 engagement imparted a pro-inflammatory M1-skewed polarization state to hypoxic macrophages [31]. 

In vivo, TREM-1 was first identified in sepsis and suggested to be an important diagnostic/prognostic marker in both bacterial and fungal infections. The release of elevated levels of sTREM-1 has been observed in serum samples from septic patients [162,168,184], as well as in biologic fluids and inflammatory lesions from patients with lower respiratory tract and pleural infections [37,154,157,212,213]. The TREM-1 role as an amplifier of inflammatory responses was confirmed in experimental models of septic shock and other infectious conditions. Recent studies described the TREM-1 involvement in viral and parasite-associated infections and its potential as a biomarker in these diseases [181,189,192]. The pharmacologic inhibition of TREM-1 signaling by administration of the TREM-1/Fc fusion protein or siRNAs partially protected animals from acute inflammation and death induced by microbial TLR ligands [37,154,161,162,168,181,186,214,215,216].

More recent investigations have linked TREM-1 to the occurrence of acute non-infectious inflammatory diseases, such as acute pancreatitis, myocardial ischemia–reperfusion injury, and hemorrhagic shock, as well as to the perpetuation of several chronic inflammatory conditions, including bowel inflammatory disorders, arthritides, atherosclerosis, hypertrophic scars/ulcers, multiple sclerosis, chronic obstructive pulmonary disease, and chronic hepatic granulomatous inflammation [37,110,153,157,178,217,218,219,220]. Increased TREM-1 expression levels and sTREM concentrations in various biological fluids were associated with early organ inflammatory injury and dysfunction and suggested to be an indicator of disease activity and severity. This issue is addressed in detail in Reference [37].

## 6. TREM-1 Role in Cancer

Recent investigations using different modulators of TREM-1 signaling have highlighted its critical involvement in inflammation-mediated carcinogenesis (Figure 2). High TREM-1 expression on MPs in mouse and human tumors and/or sTREM-1 release into the biologic fluids of cancer patients were indicated as independent predictors of tumor progression and poor patient prognosis [37,160]. (Table 1). 

Growing evidence points to a causal role of TREM-1 in chronic inflammation-mediated lung cancer. In normal pulmonary tissue, TREM-1 is selectively expressed in alveolar macrophages, which specialize in pathogen and apoptotic cell clearance. Analysis of surgical specimens from NSCLC patients demonstrated a high number of TREM-1+ macrophages in tumor tissues and pleural effusions, that was associated with elevated sTREM-1 concentrations and correlated with aggressive tumor behavior, recurrence, and poor patient survival, suggesting that TREM-1+ macrophages are critical players in NSCLC development and progression [194,221,222]. Lung cancer cells directly promoted TREM-1 upregulation, sTREM-1 release, and pro-inflammatory cytokine secretion in primary Mn/macrophages from NSCLC patients [194,221]. TREM-1 involvement in NSCLC progression was further demonstrated by the finding that TREM-1 engagement by an agonist mAb in macrophages increased NSCLC’s cell invasive ability, which was suppressed upon TREM-1 inhibition by shRNA [221]. TREM-1+ DCs accumulation in pleural effusions from patients with lung adenocarcinoma also seems to be associated with disease aggressiveness and bad prognosis [223]. 

IBDs are a group of chronic gastrointestinal disorders caused by genetics and environmental factors and characterized by bowel chronic inflammation associated with increased risk of colorectal and small bowel cancers [234]. Severe and diffuse mucosal inflammation develops with the production of inflammatory mediators and ulceration. TREM-1 appears to be crucially implicated in IBD pathogenesis and progression to colon tumorigenesis [224,225]. In normal human intestine, only a small fraction of resident macrophages express TREM-1, probably because local IL-10 and TGFβ prevent excessive activation in response to the flora-rich microenvironment [193]. In contrast, in the mucosa of IBD patients and experimental models of colitis, TREM-1-expressing macrophage infiltration becomes markedly increased, amplifying intestinal inflammation and tissue damage through the secretion of pro-inflammatory mediators [224]. TREM-1-deficiency in mouse models of colitis significantly attenuates disease severity, reduces pro-inflammatory cytokine production and inflammatory infiltrates, and prevents colon carcinoma development/progression, pointing to a pro-oncogenic role of TREM-1 through the amplification of MP inflammatory responses [226]. In addition, increased plasma sTREM concentrations were found in IBD patients [224], representing an indicator of clinical activity [225,227]. TREM-1 pharmacologic blockade in vivo in mouse models of colitis confirmed the TREM-1 contribution to the exacerbation and perpetuation of chronic colon inflammation and its progression toward carcinoma development [235] (see Section 7 for details).

TREM-1 was also indicated as a pivotal determinant of HCC development, progression, and poor prognosis [208]. HCC is a well-known inflammation-related cancer, whose pathogenesis is mediated by Kupffer cells in conjunction with recruited inflammatory cells which produce high amounts of inflammatory cytokines after hepatocyte death in the setting of hepatitis or cirrhosis, driving the compensatory proliferation of remaining hepatocytes that ultimately evolves into HCC [228]. TREM-1 mRNA and protein upregulation in liver Kupffer cells was associated with an increased inflammatory response, hepatic injury, and HCC development in a murine model of hepatocellular carcinogenesis, that were attenuated in TREM-1-deficient mice [208]. Another study showed an elevated TREM-1 expression in human HCC-activated hepatic stellate cells (HSCs) from peri-tumoral specimens, associated with HCC aggressive behavior and poor patient survival, serving as an independent prognostic predictor for both early tumor recurrence and low patient overall survival [229]. The sTREM-1 level was higher in the plasma of HCC patients than in those with benign liver tumors [229], suggesting its relevance as a tumor biomarker. Duan et al. [230] further demonstrated a significant correlation among a high TREM-1 expression, pro-inflammatory cytokine secretion, increased HCC proliferation/migration, and decreased HCC patient survival, suggesting a potential prognostic value for postoperative recurrence. Interestingly, a recent study demonstrated that a novel small-molecule STAT3-inhibitor able to block liver inflammation and reduce HCC development/growth in a mouse model of nonalcoholic steatohepatitis exerted a main inhibitory effect on TREM-1 signaling, further linking TREM-1 to the inflammatory processes that promote liver cancer [231]. Finally, Wu et al. [205] recently demonstrated that TREM-1^+^ TAMs were abundant in advanced-stage HCC within HIF+ areas and associated with poor prognosis, and that, in orthotopic liver tumor models, TREM-1+ TAMs induced immunosuppression by impairing CD8+T cell cytotoxicity while promoting their apoptosis. Interestingly, PD-L1 blockade failed to improve TREM-1+TAM-mediated immunosuppression in tumor-bearing mice because of Treg recruitment by the TREM-1-induced CCL20 release in response to the hypoxic environment [205]. 

TREM-1 upregulation on blood Mn and TAMs phenotypically and functionally resembling myeloid-derived suppressor cells (MDSCs) was reported in mice bearing s.c. T-cell lymphomas, correlating with an increased tumor volume, suggesting its potential contribution to MDSC immune suppressive activity [182].

Finally, a critical role for TREM-1-expressing TAMs in pancreatic cancer progression was shown in xenograft mouse models [232] (see Section 7 for details), and a TREM-1 increased expression was also observed in Mn from patients with invasive cervical cancer [233] and in TAM infiltrating glioblastoma [202].

## 7. TREM-1 Therapeutic Targeting 

The evidence that TREM-1 blockade via the TREM-1/Fc protein/antagonist Abs, or genetic deletion/silencing can attenuate inflammatory responses and disease severity in experimental models of microbial infection and inflammation, while treatment with agonist mAbs worsened the disease, indicated the potential of this molecule as a therapeutic target in inflammatory conditions [37,185,191,220,226]. Many efforts have therefore been focused on the development of inhibitors targeting different TREM-1 domains to prevent receptor activation and function [37,157,178,236]. Two short synthetic peptides, P1 and LP17, derived from the TREM-1 ligand-binding extracellular domain and behaving as decoys for endogenous TREM-1 ligands, have been created and proven to be effective in various preclinical models of microbial infections/sepsis and non-infectious inflammatory conditions by dampening inflammation and protecting from organ damage and death [37,168,178,184,186,188]. A ligand-independent peptide inhibitor, GF9, targeting the TREM-1 transmembrane domain and preventing the TREM-1/DAP12 interaction [237], has shown therapeutic efficacy in models of septic shock [37]. Relevant preclinical data have also been obtained using the TREM-1 antagonist peptide, LR12, mimicking a conserved sequence across TREM-1 and the related receptor, TREM-like transcripts-1 [238], in animal models of septic shock and myocardial ischemia (for a review see Reference [37]).

Recently, experimental and human studies have suggested that TREM-1 pharmacologic targeting may represent a potential novel therapeutic modality to inhibit MP-mediated chronic inflammation-associated tumor development, pointing to TREM-1 as an attractive new immunotherapeutic target also for cancer [37,157] (Figure 2, Table 2). 

Zhou and coworkers [235] demonstrated that TREM-1 blocking by the administration of the LP17 peptide antagonist had therapeutic effects in a mouse model of colon carcinogenesis, reducing the histopathological alterations and tumor formation associated with decreased levels of pro-inflammatory cytokine production by intestinal macrophages and epithelial proliferation, suggesting that treatment can attenuate intestinal macrophage-mediated inflammation and progression to colon carcinoma [235]. 

The GF9 effects were explored in two human NSCLC xenograft models. TREM-1 blockade by the administration of the GF9 inhibitory peptide significantly suppressed tumor growth, and the therapeutic efficacy was increased by the peptide incorporation into synthetic high-density lipoprotein nanoparticles to extend its half-life and specifically target delivery to macrophages, supporting the TREM-1 potential as a target for adjunctive therapy in lung cancer treatment [237]. The GF9 peptide also showed therapeutic potential against pancreatic cancer. Its administration in three human pancreatic cancer xenograft mouse models resulted in a strong anti-tumor effect, which was significantly correlated with the suppression of TAM infiltration, reduction of pro-inflammatory cytokine serum levels, and increased animal survival [232]. In addition, blocking TREM-1 signaling with GF9 treatment in orthotopic HCC-bearing models abrogated the TREM-1^+^TAM-mediated immunosuppressive effects by significantly reducing Treg recruitment and CD8^+^T cell apoptosis/dysfunction, showing inhibitory effects on tumor growth and improving mouse survival [205]. Interestingly, GF9 treatment also significantly attenuated resistance to PD-L1 blockade, improving its therapeutic efficacy [205]. 

These data suggest that TREM-1-specific peptide inhibitors have a cancer type-independent anti-tumor activity and can potentially be used as a stand-alone therapy or as a component of combinational therapy for several types of solid tumors.

## 8. Concluding Remarks

In this review, we have summarized some general trends emerging from the published data concerning MPs’ role in the inflammatory circuit that promotes tumor progression and their potential as effectors of cancer immunotherapy. Outlined studies emphasize the complex level of control exerted by PRRs and IRS receptors expressed on MPs on the development and perpetuation of chronic inflammatory conditions that predispose patients to cancer and highlight the promises of their targeting with specific mAbs or agonist/antagonist ligands to reprogram immunosuppressive/ pro-tumoral MPs toward an anti-tumor direction. A clear conclusion is that MP receptor-targeted immunotherapeutic approaches have the potential to complement and synergize with current treatments to improve their efficacy. The identification of novel targets of treatment would be helpful to create new combination anti-cancer therapies. 

The TREM-1 pharmacological inhibition with specific peptide inhibitors has recently proven to be effective in various mouse models of chronic inflammation-associated malignancies, conferring protection from tumor growth and survival advantages through the attenuation of MP inflammatory responses, pointing to TREM-1 as a novel attractive target for clinical application in cancer. The advantage of such an approach is that it blunts, but does not totally abrogate, inflammatory responses, which are essential for tumor control. So far, however, the therapeutic use of TREM-1 inhibitors has been limited to preclinical models, and only studies designed to measure the prognostic/diagnostic value of TREM-1 expression and/or sTREM-1 levels in samples from patients with inflammation-associated cancer have been carried out [37,157]. Recently, a phase I clinical trial has been run to evaluate the safety, tolerability, and pharmacokinetics of the synthetic peptide nangibotide (LR12), the first drug candidate targeting TREM-1 to reach clinical stage development [239], and future assessment of this agent for cancer therapy is expected. 

Therapeutic applications directed to TREM-1 revolve around antagonizing its function. Small synthetic peptide antagonists have been widely tested because of their low immunogenicity [157]. The TREM-1/Fc fusion protein has emerged by studies in microbial sepsis [162,240,241] and other inflammatory conditions [185,220,242,243] as an alternative TREM-1 inhibitor, potentially able to overcome the limited pharmacological effects of synthetic peptides in humans due to their short half-life. Studies to derive new peptides or small molecules able to counteract the TREM-1 pro-tumoral effects, but also to activate innate immunity via TREM-1/DAP12 pathways in the context of vaccination, are currently ongoing (for a review see Reference [157]). Clinical testing of antagonist anti-TREM-1 Abs is likewise warranted because Abs bind directly to TREM-1, whereas peptides might only bind to its ligands or DAP12 (Table 2). A better understanding of the identity of the TREM-1 ligand might reveal novel targets for tumor treatment. 

Further research is crucial before immunotherapeutic strategies based on TREM-1 targeting could be translated into clinical settings. Divergent results were in fact reported by Zhang et al. [244], who showed that TREM-1 expression in TAMs and Mn were decreased during lung tumor progression. An additional level of complexity is given by the evidence of TREM-1 expression on other myeloid and non-myeloid cells involved in anti-tumor responses [37]. TREM-1 is highly expressed in neutrophils, where its activation results in ROS, lactoferrin, myeloperoxidase, and NO production, with consequent increased neutrophil degranulation, oxidative burst, and phagocytosis [45,156,173]. In addition, this molecule has been recently reported to be required for successful NK cell anti-tumor activity in a mouse lung tumor model. Lee et al. [245] demonstrated that lung tumor development and metastasis were suppressed via an increase in TREM-1-dependent NK cell cytotoxicity. TREM-1 blockade with LP-17 prevented NK cell killing, whereas TREM-1 mAb recovered NK cytotoxic effects. Under this scenario, the inhibition of TREM-1 would lead to increased tumor growth. Therapeutic effects of TREM-1 targeting will thus be ultimately dictated by the functional interplay among the different TREM-1+ immune cell populations recruited to tumor sites, suggesting that caution should be exerted in modulating its activity. 

Finally, evidence linking low pO_2_, HIF-1 expression, deregulated MP receptor expression/activities, and MP pro-tumoral activation at tumor sites have potential implications for the design of new HIF-1-targeted therapies (Figure 2). The inhibition of hypoxia-mediated tumor promotion by blocking the HIF-1 expression/activity is currently being investigated as a therapeutic approach for various malignancies [91]. Several putative small molecule HIF-1 inhibitors have been tested in clinical trials [82,246,247] and may have potential efficacy in counteracting MP receptor deregulation and promoting MP anti-tumor responses.

## Figures and Tables

**Table 1:** TREM-1 mRNA/protein expression in mouse and human tumor-associated MPs and/or sTREM-1 release into the biological fluids of cancer patients.

Cancer Type	MP Type	Biological Fluid	Effects	References
NSCLC	Macrophages in tumors and pleural effusions	Pleural effusions	Correlates with aggressive tumor behavior, recurrence, poor disease-free, and overall patient survival	[194,221,222]
Lung adenocarcinoma	DCs in pleural effusions	ND	Associates with disease aggressiveness and bad prognosis	[223]
Colon carcinoma	Intestinal macrophages	Serum/plasma	Amplifies macrophage- mediated inflammation and intestinal tissue damage; correlates with aggressive tumor behavior and recurrence	[224,225,226,227]
HCC	Kupffer cells; HSCs; macrophages in tumor specimens	Plasma	Increases inflammatory responses, hepatic injury, tumor development and aggressive behavior, and poor patient survival; prognostic predictor for both early tumor recurrence and low patient overall and relapse-free survival after resection; mediates immunosupression	[205,208,228,229,230,231]
Pancreatic cancer	TAMs	ND	Correlates with cancer progression	[232]
T-cell lymphoma	MDSCs; TAMs	ND	Correlates with increased tumor volume	[182]
Cervical cancer	Monocytes	ND	Correlates with high-grade, invasive cancer	[233]
Glioblastoma	TAMs	ND	Associates with low outcome of chemotherapy-treated patients	[202]

Abbreviations: NSCLC, non-small cell lung cancer; HCCs, hepatocellular carcinoma; HSCs, HCC-activated hepatic stellate cells; TAMs, tumor-associated macrophages; MDSC, myeloid-derived suppressor cells; ND, not determined.

**Table 2:** Therapeutic effects of TREM-1 targeting by synthetic peptide inhibitors in preclinical mouse models of inflammation-derived malignancies.

Cancer Type	TREM-1 Peptide	Therapeutic Effects	References
Colon Cancer	LP-17	Reduces pro-inflammatory mediator secretion by intestinal macrophages; attenuates intestinal inflammation, permeability, and epithelial damage; decreases epithelial histopathological alterations, proliferative activity, and progression to colon carcinoma	[235]
NSCLC	GF9	Decreases cytokine production and delays tumor growth	[237]
Pancreatic Cancer	GF9	Reduces TAMs infiltration, pro-inflammatory cytokine serum levels, and tumor growth, increasing animal survival	[232]
HCC	GF9	Reduces tumor development and growth; abrogates TREM-1+TAM-mediated immunosuppressive effect by reducing Treg recruitment and CD8+T cell apoptosis/dysfunction; improves mouse survival; attenuates resistance to PD-L1 blockade improving its therapeutic efficacy	[205]

Abbreviations: NSCLC, non-small cell lung cancer; HCC, hepatocellular carcinoma; TAMs, tumor-associated macrophages; PD-L1, programmed cell death-1 ligand.

## Abbreviations

TME	tumor microenvironment
IBD	inflammatory bowels disease
MPs	mononuclear phagocytes
PAMPs/DAMPs	pathogen- or damage-associated molecular patterns
TREM 1	triggering receptor expressed on myeloid cells
Mn	monocytes
TAMs	tumor-associated macrophages
TADCs	tumor-associated dendritic cells
ROS	reactive oxygen species
NO	nitric oxide
pO2	partial oxygen pressure
PRRs	pattern recognition receptors
IRS	immunoregulatory signaling
ADCC	Ab-dependent cell-mediated cytotoxicity
TLRs	toll-like receptors
MSR	macrophage scavenger receptor 1
MARCO	macrophage receptor with collagenous structure
NSCLC	non-small cell lung cancer
SIRPα	signal regulatory protein-alpha
CD47	cluster of differentiation 47
PD1	programmed cell death-1
PDL1	programmed cell death-1 ligand
LILRB1	leukocyte immunoglobulin-like receptor B1
β2M	β2-microglobulin
TIM-3	T cell Ig and mucin domain 3
HMGB1	high mobility group box 1
mAbs	monoclonal antibodies
FcRs	Fc region of Igs
ADP	Ab-dependent phagocytosis
CYT	cytoplasmic tail
DAP12	DNA activating protein 12
ITAM	immunoreceptor tyrosine-based activatory motif
sTREM-1	soluble TREM-1
MMP	metalloproteinase
LCs	Langerhans cells
HRE	hypoxia response element
HIF1	hypoxia-inducible transcription factor
HCC	hepatocellular carcinoma
siRNA	small interfering RNA
HSCs	hepatic stellate cells
